# Cascade process mediated by left hippocampus and left superior frontal gyrus affects relationship between aging and cognitive dysfunction

**DOI:** 10.1186/s12868-021-00680-x

**Published:** 2021-12-07

**Authors:** Yumika Kokudai, Motoyasu Honma, Yuri Masaoka, Masaki Yoshida, Haruko Sugiyama, Akira Yoshikawa, Nobuyoshi Koiwa, Satomi Kubota, Natsuko Iizuka, Sayaka Wada, Shotaro Kamijo, Yuki Uchida, Satoshi Yano, Masahiro Ida, Kenjiro Ono, Masahiko Izumizaki

**Affiliations:** 1 Department of Physiology, Showa University School of Medicine, 1-5-8 Hatanodai, Shinagawa-ku, Tokyo, 142-8555 Japan; 2 Department of Ophthalmology, Jikei University School of Medicine, Tokyo, Japan; 3 Sensory Science Research, Kao Corporation, Tokyo, Japan; 4 School of Nursing and Rehabilitation Sciences, Showa University, Yokohama, Japan; 5 Human Arts and Sciences Research Center, University of Human Arts and Sciences, Saitama, Japan; 6 Division of Neurology, Department of Medicine, Showa University School of Medicine, Tokyo, Japan; 7 Department of Radiology, Stroke Center, Ebara Tokyo Hospital, Tokyo, Japan; 8 Department of Neurology and Neurobiology of Aging, Kanazawa University Graduate School of Medical Sciences, Kanazawa, Japan

**Keywords:** Cognitive dysfunction, Wisconsin card sorting test, Aging, Hippocampus, Superior frontal gyrus

## Abstract

**Background:**

Cognitive function declines with age and has been shown to be associated with atrophy in some brain regions, including the prefrontal cortex. However, the details of the relationship between aging and cognitive dysfunction are not well understood.

**Methods:**

Across a wide range of ages (24- to 85-years-old), this research measured the gray matter volume of structural magnetic resonance imaging data in 39 participants, while some brain regions were set as mediator variables to assess the cascade process between aging and cognitive dysfunction in a path analysis.

**Results:**

Path analysis showed that age affected the left hippocampus, thereby directly affecting the left superior frontal gyrus. Furthermore, the gyrus directly affected higher order flexibility and maintenance abilities calculated as in the Wisconsin card sorting test, and the two abilities affected the assessment of general cognitive function.

**Conclusion:**

Our finding suggests that a cascade process mediated by the left hippocampus and left superior frontal gyrus is involved in the relationship between aging and cognitive dysfunction.

**Supplementary Information:**

The online version contains supplementary material available at 10.1186/s12868-021-00680-x.

## Introduction

Higher brain functions related to thinking, judgment, and behavior are highly human-specific characteristics. In particular, executive functioning to tune out stimuli that are irrelevant to the task/process (cognitive inhibition) or to adapt strategies to situations (cognitive flexibility), as assessed by the Wisconsin card sorting test (WCST) [1] and Montreal Cognitive Assessment (MoCA) [2], which measures overall cognitive function, plays an important role in daily life [3–5]. Although the executive functioning declines with age [6], it is also affected by years of education and illness [7–9], making individual differences to be quite pronounced [10].

Executive functioning is associated with the hippocampal-frontal network [11–13], and some studies suggest that the volume and thickness of the frontal lobe and hippocampus are involved in performance in the WCST [14, 15]. In addition, positron emission tomography and electroencephalography patterns during the WCST are also affected by aging [16, 17]. It is thought that the atrophy of specific regions is an intervening variable in the relationship between aging and the decline in executive functioning; however, the details of the cascade process among relevant variables have not been elucidated to date.

The current study measured the gray matter volume from structural magnetic resonance imaging (MRI) data, and the frontal lobe and hippocampus were set as regions of interest (ROIs) (12 locations on the left and right sides of the ROIs: the opercular part of the inferior frontal gyrus, orbital part of the inferior frontal gyrus, triangular part of inferior frontal gyrus, middle frontal gyrus, superior frontal gyrus, and hippocampus) because they were related to WCST in previous studies mentioned above [11–17]. First, we assessed the decline in executive functioning (WCST and MoCA) and region atrophy due to aging by comparing younger and older groups. Next, we assessed the relationship between age, years of education, region atrophy, WCST performance, and MoCA by a path analysis to verify the cascade process leading to cognitive dysfunction.

As a rough preliminary model, we assumed that individual profile characteristics would affect the volume of specific brain regions within the frontal lobe-related and hippocampal regions, and then some regions would affect cognitive function. Therefore, three phases were established in the path analysis (Additional file 1: Fig. S1). Phase 1 was set for individual profile characteristics (age and years of education), phase 2 for brain region volume (12 regions), and phase 3 for assessments of cognitive function (three indices calculated by WCST and a MoCA).

## Methods and materials

### Participants

This research was approved by the Ethics Committee of Showa University Hospital and was conducted in accordance with the principles of the Declaration of Helsinki (Clinical trial identifier number: 2561). This study was registered for the University hospital Medical Information Network (UMIN)-CTR (ID: UMIN000033776, 20/08/2018). The participants included in this study comprised a subset of subjects from a previous study [18], for whom both cognitive task and structural MRI data were available. The exclusion criteria were history of stroke, encephalitis, multiple sclerosis, history of alcohol or other drug intoxication, presence of tumors, overt sensory deficits, upper motor neuron signs, significant ball symptoms, and diffuse muscle weakness. Furthermore, patients were excluded if they had received any experimental drugs within 30 days prior to the experiment, or if they had any restrictions on MRI examinations (e.g., pacemaker, continuous infusion pump implantation, pregnant or lactating women). We also excluded those who appeared to have difficulties complying with the experiment due to mental abnormalities. All elderly participants, living independently and with self-reported absence of memory and mild cognitive deficits, participated in the study. Participants were aged 23 to 59 years in the younger group and 60 to 85 years in the elderly group. All participants provided written informed consent. Twenty elderly participants (10 men and 10 women) and 20 younger adults (11 women and 9 men) participated in this study. The average age of all participants was 56.95 (SD = 18.95). Nineteen elderly and 18 younger participants were right-hand dominant, and all had normal visual acuity. The average age of the elderly group (mean age: 74.1) was higher than that in the younger group (mean age: 40.7; *t*_37_ = 11.970, *p* < 0.0001).

### Wisconsin card sorting test

We used a modified and computerized version of the Keio Version WCST [19]. The WCST is a test that uses cards with printed figures comprising one to four triangles, stars, crosses, and circles in red, green, yellow, and blue. Participants were required to place the response cards one by one under the four stimulus cards according to one of the three classification categories: color, shape, or number. The outcome measures were the number of categories achieved (CA), perseverative errors of Nelson (PEN), and difficulties in maintaining set (DMS). The CA is the number of categories for which six consecutive correct responses are achieved (eight is the maximum number of categories that can be achieved) and reflects the sum measure of the level of conceptual shifts. The index reflects the degree of concept formation and transformation. PEN reflects the number of incorrect responses in the same category as the immediately preceding incorrect response. The index indicates a tendency for false reactions to persist and a failure to suppress the previous reaction. DMS reflects the number of false responses after consecutive correct answers. The index refers to the degree to which the subject loses track of the classification category to which the participant is conforming.

### MRI acquisition

MRI data were obtained at Ebara Hospital (Tokyo, Japan) using a Siemens Avanto 3 T Magnetom TIM Trio scanner. T1-weighted anatomical scan was performed based on the following parameters: repetition time, 2250 ms; echo time, 3.06 ms; flip angle, 9°; inversion time, 1000 ms; field of view, 256 × 256 mm; matrix size, 256 × 256; and voxel size, 1 × 1 × 1 mm. The acquisition of high-resolution anatomical images was optimized with magnetization-prepared rapid gradient echo sequence.

### Image processing

Image processing was performed using FreeSurfer version 6 [20, 21], including motion correction, removing non-brain tissue, normalization with non-uniform intensity, affine registration to Montreal Neurological Institute (MNI) space, and Talairach transformation [22]. Volumetric segmentation [23], cortical surface reconstruction [24–26], and parcellation [27, 28] were automatically performed using the recon-all script on FreeSurfer after the image processing. Detailed descriptions have been provided elsewhere [29]. The 70 gray matter volumes determined by Desikan-Killiany brain atlas [28] were used in this study. All gray matter boundaries were confirmed by visual inspections of two trained neurologists with a graphic tool of FreevVew after affine registration to MNI space. The two neurologists performed manual editing within the range of removing non-brain tissue included within the cortical boundary. Intracranial volume (ICV) was estimated using FreeSurfer version 6 [20, 21] and was used as a covariate in the statistical analysis.

The regional brain volumes were normalized using the ratio and residual methods [30]. The ratio-corrected volumes were calculated as the ratio of the regional brain volume to the ICV. For the residual method, we expressed the ICV-corrected measurements as

$$CV = V - S\left( {ICV - \overline{ICV} } \right)$$

where *CV* (Corrected Volume) is the ICV-corrected regional brain volume, *V* is the original uncorrected volume, *S* is the slope of the linear regression of *V* on ICV, ICV is the intracranial volume for a particular participant, and $\overline{ICV}$ is the mean ICV of all participants. ANCOVA was used to compare the region volumes (*CV*) between groups, with years of education and MoCA scores as covariates.

### Statistical analysis

An unpaired *t*-test was used to compare the age, years of education, and MoCA scores between the two groups. A one-way repeated measure analysis of covariance (RM-ANCOVA) was used to assess the age effect in the younger and elderly groups, with years of education and MoCA scores as covariates. We used repeated-measure ANCOVA because each participant repeated the task, and we needed to consider years of education and MoCA as covariates. Twelve regions were selected as the ROIs. Post-hoc *t*-tests with Bonferroni correction were performed for multiple comparisons for both the ANCOVA and RM-ANCOVA analyses. All tests were two-tailed. Results are presented as mean ± standard error of the mean. SPSS version 26 was used for all statistical analyses. Relationships among age, years of education, 12 ROIs (CV), 3 indices in WCST, and MoCA scores were determined using path analysis. The goodness of fit of index (GFI), root mean square error of approximation (RMSEA), comparative fit index (CFI), and Bollen-Stine bootstrap were calculated to check the model fitting. Bootstrapping is a method of randomly multiple resampling from the obtained samples and obtaining estimates from the resamples. When testing for effects in a small sample, estimation of standard errors by the bootstrap method is considered effective [31]. AMOS 27.0, was used for path analysis. Statistical significance was defined as an adjusted *p-*value of < 0.05.

## Results

One man in the elderly group was diagnosed with cerebral infarction, and his data were excluded from the analysis.

### Group comparison in the participant’s profile

The average number of years of education in the elderly group (average = 13.4, SD = 2.6, range = 9–17) was lower than that in the younger group (average = 17.1, SD = 2.4, range = 14–22) (*t*_37_ = 4.610, *p* < 0.0001). The average MoCA score in the elderly group (average = 25.8, SD = 2.2, range = 23–29) was lower than that in the younger group (average = 27.9, SD = 2.1, range = 25–30) (*t*_37_ = 3.012, *p* = 0.005).

### Group comparison in the Wisconsin card sorting test

**Fig. 1:**
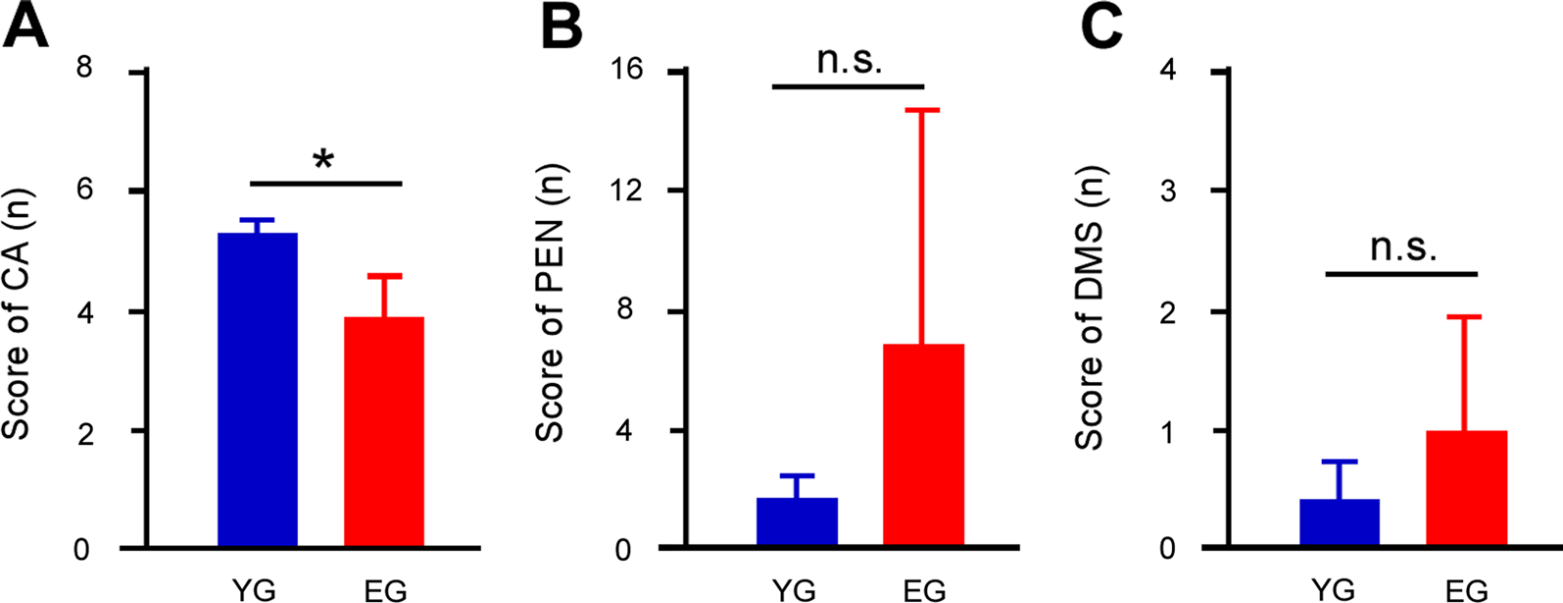
Results of Wisconsin card sorting test. **A** Scores of the categories achieved (CA) in the elderly group (EG) was lower than that in the younger group (YG). **B** Scores of the perseverative errors of Nelson (PEN) revealed no difference between the groups. **C** Scores of the difficulties of maintaining set (DMS) revealed no difference between the groups. Asterisks indicate significant differences (*p* < 0.05). Error bars indicate the standard error of mean

For the WCST, a one-way RM-ANCOVA with years of education and MoCA as covariates showed that the CA score of the elderly group was lower than that of the younger group (Fig. 1A: F_1,35_ = 5.058, *p* = 0.031), while there were no group differences in the PEN (Fig. 1B: F_1,35_ = 3.375, *p* = 0.075) or DMS scores (Fig. 1C: F_1,35_ = 0.887, *p* = 0.353).

### Group comparisons in region volumes

For the ROIs, the ANCOVA with years of education and MoCA as covariates showed that eight of the 12 regions differed significantly between the two age groups (Table 1). The elderly group had smaller volumes in the left triangular part of the inferior frontal gyrus (*p* = 0.020), left middle frontal gyrus (*p* = 0.003), left superior frontal gyrus (*p* = 0.005), left hippocampus (*p* < 0.0001), right opercular part of inferior frontal gyrus (*p* = 0.040), right middle frontal gyrus (*p* = 0.003), right superior frontal gyrus (*p* < 0.0001), and right hippocampus (*p* < 0.0001) than the younger group.

**Table 1:** Results of region volume

	Younger group		Elderly group		
	Average	S.D	Average	S.D	*p* value
Left opercular part of inferior frontal gyrus	3072	437	2946	281	0.291
Left orbital part of inferior frontal gyrus	1065	152	1028	176	0.486
Left triangular part of inferior frontal gyrus	2426	481	2217	297	0.020
Left middle frontal gyrus	9904	1076	8706	1153	0.002
Left superior frontal gyrus	17,101	1289	15,377	1401	< 0.0001
Left hippocampus	4061	287	3555	306	< 0.0001
Right opercular part of inferior frontal gyrus	3268	438	2939	442	0.025
Right orbital part of inferior frontal gyrus	986	171	912	208	0.176
Right triangular part of inferior frontal gyrus	2114	394	1980	423	0.314
Right middle frontal gyrus	8758	1026	7818	802	0.003
Right superior frontal gyrus	16,116	1431	14,202	1399	< 0.0001
Right hippocampus	4276	344	3727	325	< 0.0001

*MoCA* Montreal Cognitive Assessment, *Years of Education* Years of education since entering elementary school

The standard deviations are shown in parentheses

The unit of region volume corrected by ICV is mm^3^

Unpaired *t* test was used to group comparison

### Path analysis

**Fig. 2:**
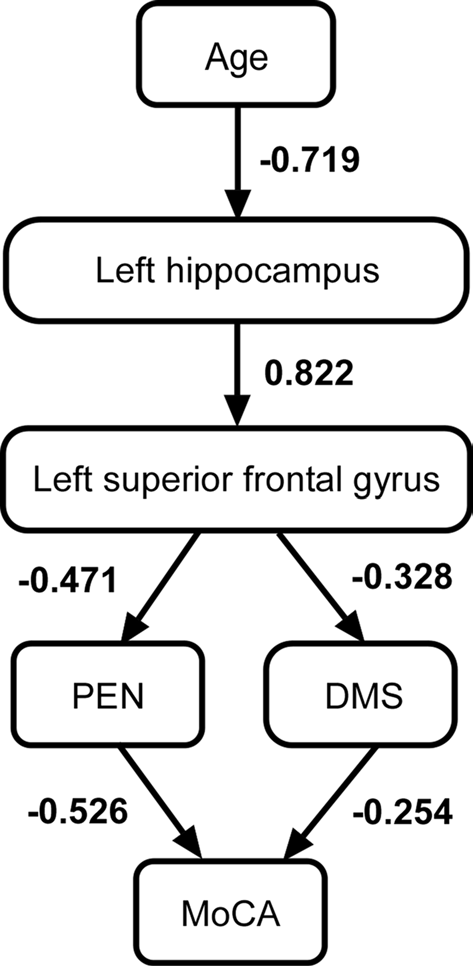
Path diagram. The age, years of education, 12 regions, 3 WCST scores, and MoCA were set as the observed variables (Additional file 1: Fig. S1). Finally, the path diagram reflected the relationship among the six variables. The solid lines indicate a significant direct effect. Numbers indicate the standardized path coefficients

We conducted a path analysis to assess the cascade process from age to overall cognitive function. Age, years of education, 12 region volumes corrected by ICV, 3 WCST scores, and MoCA scores were set as the observed variables. The most suitable model was the path in which age affected the left hippocampus (standardized path coefficient: − 0.712), left hippocampus affected the left superior frontal gyrus (0.811), left superior frontal gyrus affected PEN (− 0.464) and DMS (− 0.321), and PEN (− 0.526) and DMS (− 0.254) affected the MoCA (Fig. 2: chi- square (9) = 16.473, p = 0.058; GFI = 0.892; RMSEA = 0.148; CFI = 0.927; Bollen-Stine bootstrap = 0.174).

## Discussion

The WCST performance revealed a difference in CA, while no difference was observed in PEN and DMS between the aged groups. This may have reflected the large variance in the PEN and DMS data, suggesting that the age group comparison had no direct impact on the PEN and DMS. The CA is meant to be an overall assessment of the WCST, while the PEN reflects a response inhibition and DMS reflects attention maintenance [32]. In our results, these response inhibition and attention maintenance functions are likely to be independent of age.

**Fig. 3:**
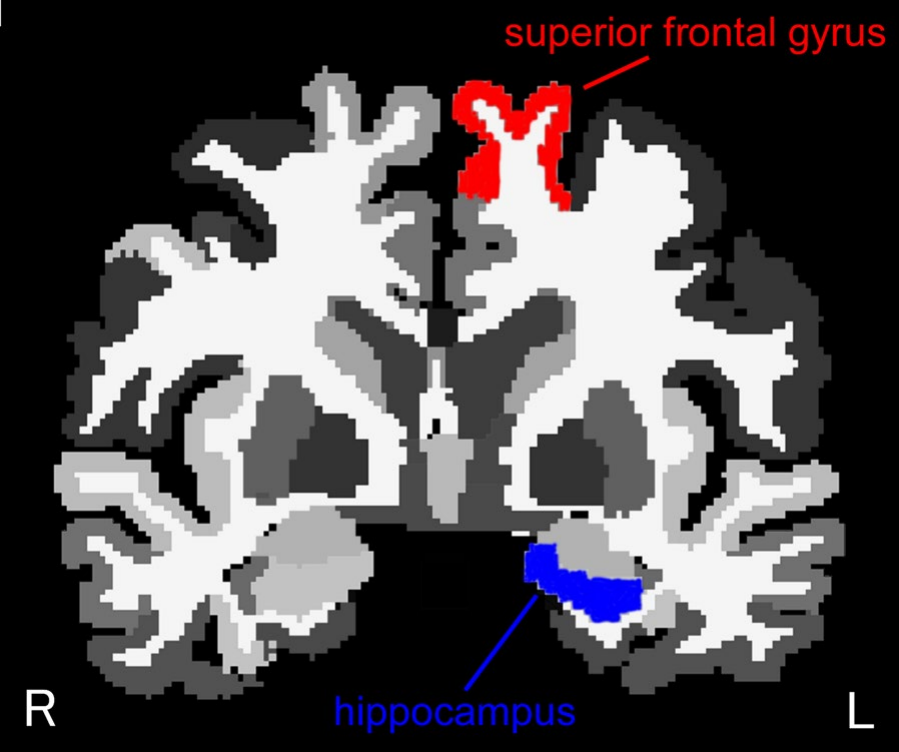
Location of left superior frontal gyrus (red) and left hippocampus (blue) in coronal view

In the age group comparison of ROIs, most regions were smaller in the elderly than in the younger group. However, in the path analysis, the left superior frontal gyrus (Fig. 3) was found to be the key intervening variable affecting WCST performance, while many ROIs were not used as the influencing variable. Furthermore, the left hippocampus, which influences the left superior frontal gyrus, was more strongly affected by age. This path analysis, which deals with the volume of brain regions, is limited in its ability to shed light on the relationship between regions. However, the hippocampus is known to be the first region associated with cognitive decline and is associated with early symptoms of Alzheimer’s disease [33]. As such, the model whereby age has the strongest and most direct effect on the hippocampus seems to be supported by previous findings. Furthermore, the model of the hippocampus influencing the WCST via the superior frontal gyrus was more suitable than the model of the hippocampus directly influencing the WCST. A reduction in the superior frontal gyrus has been associated with WCST performance in patients with schizophrenia and psychopathy patients [34–36], and these findings may support the current model.

Executive functioning is associated with the hippocampal-frontal network [11–13] and may require the functioning of both the hippocampus and prefrontal cortex. The results of the path analysis suggest that the left superior frontal gyrus is directly responsible for the response inhibition and attention maintenance functions shown by PEN and DMS. The left superior frontal gyrus is associated with cognitive inhibition [37–39]. Since the WCST requires inhibition of an action (button pressing) when it is judged to be an error, it is likely that this inhibitory function was associated with the left superior frontal gyrus. Furthermore, the left hippocampus is associated with cognitive flexibility [40, 41] and working memory for attention maintenance [42]. Because the WCST also requires these cognitive functions, it is likely that the left hippocampus was involved. It is possible that the hemispheric features related to executive functioning are reflected in the current model.

Brain asymmetry has long been discussed, with the left hemisphere being associated with linguistic functions (ex. Wernicke's and Brocker's language areas) [43, 44] and the right hemisphere with non-linguistic functions (ex. Flor Henry's association of temporal epilepsy with schizophrenic psychosis or affective psychosis) [45, 46]. Although WSCT is a non-linguistic function, it requires cognitive inhibition and flexibility, which are associated with the left hemisphere [37–41]. Therefore, it may be difficult to completely separate verbal and nonverbal functions in the right and left hemispheres.

The current results, although limited, suggest that when assuming a cascade process relationship between aging and cognitive decline, aging directly affected the left hemisphere (left hippocampus and superior frontal gyrus), which affected part of WSCT. These findings are consistent with those of previous studies that examined the relationship between cognitive function and brain regions [37–41]. In addition, the finding that the left hemisphere did not directly affect MoCA may be because MOCA encompasses all cognitive functions, making it less relevant to the left hemisphere. Since MoCA is an indicator of overall cognitive function, it was perhaps not surprising that PEN and DMS had a direct effect on MoCA. However, since CA did not lead to MoCA, it could be due to small variations in CA; as such, the variation may not be suitable for variations in MoCA. In fact, in the path analysis, the model fit index increased when the CA was removed.

However, the current research has some limitations. First, amyloid-β or Lewy bodies were not measured. Therefore, it is impossible to determine whether cognitive decline in the elderly was due to degenerative or vascular reasons. Future research should investigate the causes of cognitive decline and examine the relationship between cognitive function and age. Second, participants had a large difference in the number of years of education depending on age. In Japan, the college-going rate 20 years ago was twice as much that of 50 years ago, and this inter-age factor may have caused the inter-group difference in years of education. Since several studies have already shown that years of education affect cognitive function [47, 48], further investigations are needed to determine how age and years of education affect cognitive decline in elderly people. Finally, the study used a small sample. Although further research using a large sample is warranted to verify the findings of the present study, the finding that a specific brain region mediates the relationship between aging and cognitive function is a valuable insight for understanding the effects of aging.

This study found that a specific brain region mediates the relationship between age and cognitive dysfunction, which offers valuable insights into the individual differences in aging. Some people maintain their executive functioning even when they age, while others have low executive functioning even when they are young. In light of these individual differences, in the path of the relationship between age and cognitive dysfunction, we provide one explanation for the cascade process, whereby the lack of any signs of atrophy of specific brain regions may help maintain cognitive function.

## Supplementary Information

**Additional file 1:** **Figure S1. **The process of variable-setting in the path analysis. Phase 1 was set for individual profile characteristics (age and years of education), Phase 2 for brain region volume (12 regions: Left opercular part of inferior frontal gyrus, Left orbital part of inferior frontal gyrus, Left triangular part of inferior frontal gyrus, Left middle frontal gyrus, Left superior frontal gyrus, Left hippocampus, Right opercular part of inferior frontal gyrus, Right orbital part of inferior frontal gyrus, Right triangular part of inferior frontal gyrus, Right middle frontal gyrus, Right superior frontal gyrus, and Right hippocampus), and Phase 3 for cognitive function assessment (CA, PEN, and DMS calculated by WCST and MoCA).

## Data Availability

The data analyzed for the current study are not publicly available because we did not obtain the consent of participants to provide them to third parties, but the data is available from the corresponding author on reasonable request.
