# Supplementary material for: Cascade process mediated by left hippocampus and left superior frontal gyrus affects relationship between aging and cognitive dysfunction
Source: BMC Neurosci. 2021 Dec 7;22:75. doi: 10.1186/s12868-021-00680-x (PMC8650545; doi:10.1186/s12868-021-00680-x)
Supplement: Supplementary file 1 — Additional file 1: Figure S1. The process of variable-setting in the path analysis. Phase 1 was set for individual profile characteristics (age and years of education), Phase 2 for brain region volume (12 regions: Left opercular part of inferior frontal gyrus, Left orbital part of inferior frontal gyrus, Left triangular part of inferior frontal gyrus, Left middle frontal gyrus, Left superior frontal gyrus, Left hippocampus, Right opercular part of inferior frontal gyrus, Right orbital part of inferior frontal gyrus, Right triangular part of inferior frontal gyrus, Right middle frontal gyrus, Right superior frontal gyrus, and Right hippocampus), and Phase 3 for cognitive function assessment (CA, PEN, and DMS calculated by WCST and MoCA). [file 12868_2021_680_MOESM1_ESM.docx]

**Supplementary Information**

Cascade process mediated by left hippocampus and left superior frontal gyrus affects relationship between aging and cognitive dysfunction

Yumika Kokudai, Motoyasu Honma, Yuri Masaoka, Masaki Yoshida, Haruko Sugiyama, Akira Yoshikawa, Nobuyoshi Koiwa, Satomi Kubota, Natsuko Iizuka, Sayaka Wada, Shotaro Kamijo, Yuki Uchida, Satoshi Yano, Masahiro Ida, Kenjiro Ono, and Masahiko Izumizaki

**Supplementary Figure 1.**


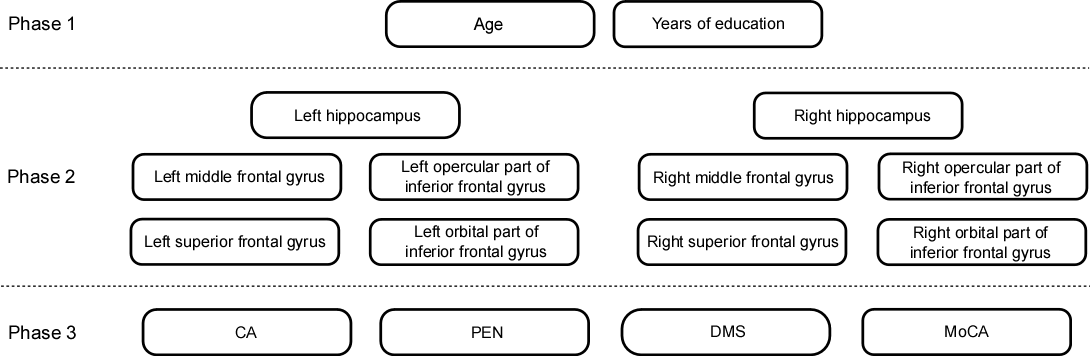


**Supplementary Figure 1.** The process of variable-setting in the path analysis. Phase 1 was set for individual profile characteristics (age and years of education), Phase 2 for brain region volume (12 regions: Left opercular part of inferior frontal gyrus, Left orbital part of inferior frontal gyrus, Left triangular part of inferior frontal gyrus, Left middle frontal gyrus, Left superior frontal gyrus, Left hippocampus, Right opercular part of inferior frontal gyrus, Right orbital part of inferior frontal gyrus, Right triangular part of inferior frontal gyrus, Right middle frontal gyrus, Right superior frontal gyrus, and Right hippocampus), and Phase 3 for cognitive function assessment (CA, PEN, and DMS calculated by WCST and MoCA).
